# Tough and Temperature‐Resistant Material Based on *Bombyx mori* Silk Fibroin

**DOI:** 10.1002/advs.202520165

**Published:** 2026-01-15

**Authors:** Meng Zhang, Quan Wan, Yajun Shuai, Qi Wu, Jing Yu, Mingzheng Fang, Yuqing Zhang, Chuanbin Mao, Mingying Yang

**Affiliations:** ^1^ Key Laboratory of Silkworm and Bee Resource Utilization and Innovation of Zhejiang Province Institute of Applied Bioresource Research College of Animal Sciences Zhejiang University Hangzhou China; ^2^ Silk Biotechnology Laboratory School of Biology and Basic Medical Sciences Soochow University Suzhou China; ^3^ Department of Biomedical Engineering The Chinese University of Hong Kong Sha Tin Hong Kong SAR China

**Keywords:** *Bombyx mori*, crystallization, hydration, silk fibroin, temperature‐resistant

## Abstract

Extreme temperatures present significant challenges for materials in polar and extraterrestrial exploration. Inspired by *Bombyx mori* spinning, we propose a biomimetic “Hydration‐Crystallization Locking” (HCL) strategy. By precisely controlling the composition and distribution of hydrophilic domains and hydrophobic crystals in silk fibroin (SF), we developed a flexible fibroin membrane (FFM) exhibiting high tensile strength (∼50.5 ± 3.2 MPa), toughness (∼16.4 ± 1.2 MJ/m^3^), and >95% shape retention under large deformations at −196°C and 70°C. The HCL strategy was confirmed to promote the formation and retention of hydrophilic Silk I structure, with its conformational signature type II β‐turn retained from 30.8% to 14.6%, while partially transitioning to the hydrophobic Silk II structure. Silk I captured immobile water via serine, forming orderly hydrated structures that enhanced chain plasticization; uniformly dispersed Silk II crystalline domains acted as hydrophobic and thermal barriers, preventing water escape and freezing. FFM served as a multifunctional platform in extreme environments for flexible photovoltaics, polar equipment, and a lightweight electromagnetic interference shielding shell. Additionally, FFM was recyclable under mild conditions. The HCL strategy enables renewable SF to replace petroleum‐based polymers for balancing mechanical properties and temperature resistance. It provides a sustainable framework for designing high‐performance biomass polymers for extraterrestrial exploration and low‐temperature systems.

## Graphical abstract

We propose a biomimetic “Hydration‐Crystallization Locking” (HCL) strategy inspired by *Bombyx mori* spinning to enhance silk fibroin (SF) performance in extreme temperatures. A flexible fibroin membrane (FFM) shows high strength, toughness, and shape retention under −196°C and 70°C. The HCL strategy optimizes hydrophilic and hydrophobic domains of SF, making FFM suitable for polar/extraterrestrial applications, offering an eco‐friendly alternative to petroleum‐based polymers.

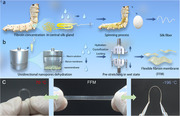

## 1 Introduction

The relentless human endeavors in polar expeditions and extraterrestrial exploration (e.g., Antarctic research stations and lunar base programs) have exposed a critical technological barrier: catastrophic material failure under extreme temperatures [1]. Conventional engineering materials undergo irreversible physicochemical degradation under such conditions, leading to structural collapse of load‐bearing components and functional deterioration of precision instruments, which directly threaten mission‐critical operations in these frontier domains [2, 3, 4]. These challenges impose stringent demands on material development.

Current material systems face significant challenges in resolving the “stability‐flexibility dilemma”. High‐performance ceramics and metal alloys [5, 6, 7, 8], although thermally stable, are hindered by intrinsic brittleness and high density, further exacerbated by dependence on non‐renewable resources. Polymer‐based materials provide lighter alternatives but show unreliable strength when exposed to temperature extremes‐either softening at high temperatures or becoming brittle in freezing conditions [9]. Recent advancements like nanoparticle‐reinforced polymers and bioinspired aerogels still struggle to balance the key requirement of how to maintain structural strength while preserving flexibility at low temperatures [10, 11, 12, 13]. The obstacle to this issue is due to the fundamental conflict between molecular rigidity and movement. Therefore, for overcoming this limitation, it is urgent to develop eco‐friendly materials that can balance molecular rigidity and movement to withstand harsh environments without compromising performance.

Interestingly, the evolutionary formation of silkworm cocoons offers a biomimetic solution to resolve the “stability‐flexibility dilemma” [14, 15, 16]. Silk fiber spun by *Bombyx mori* (*B. mori*) is one of the strongest and toughest materials produced by nature [17, 18, 19]. The exceptional mechanical property of silk fiber is contributed by silk fibroin (SF), which is a protein featuring periodically alternating hydrophilic amorphous regions and hydrophobic β‐sheet crystalline domains [20, 21]. This dual‐phase architecture enables molecular‐level reinforcement to pack into crystalline regions while maintaining molecular mobility via hydrated amorphous regions for achieving the remarkable mechanical strength and environmental tolerance [22, 23, 24, 25]. Materials based on SF have emerged as promising candidates for extreme environments because of their inherent unique structural advantages [26, 27, 28, 29]. However, there are difficulties in the processing of SF materials, such as the rapid dehydration‐induced brittleness at high temperatures and the disordered crystallization‐induced mechanical failure at low temperatures [30, 31, 32, 33]. Therefore, it is urgent to invent a way to control the interactions between the SF molecule and water to develop high‐performance SF materials for application in extreme environments.

**SCHEME 1:**
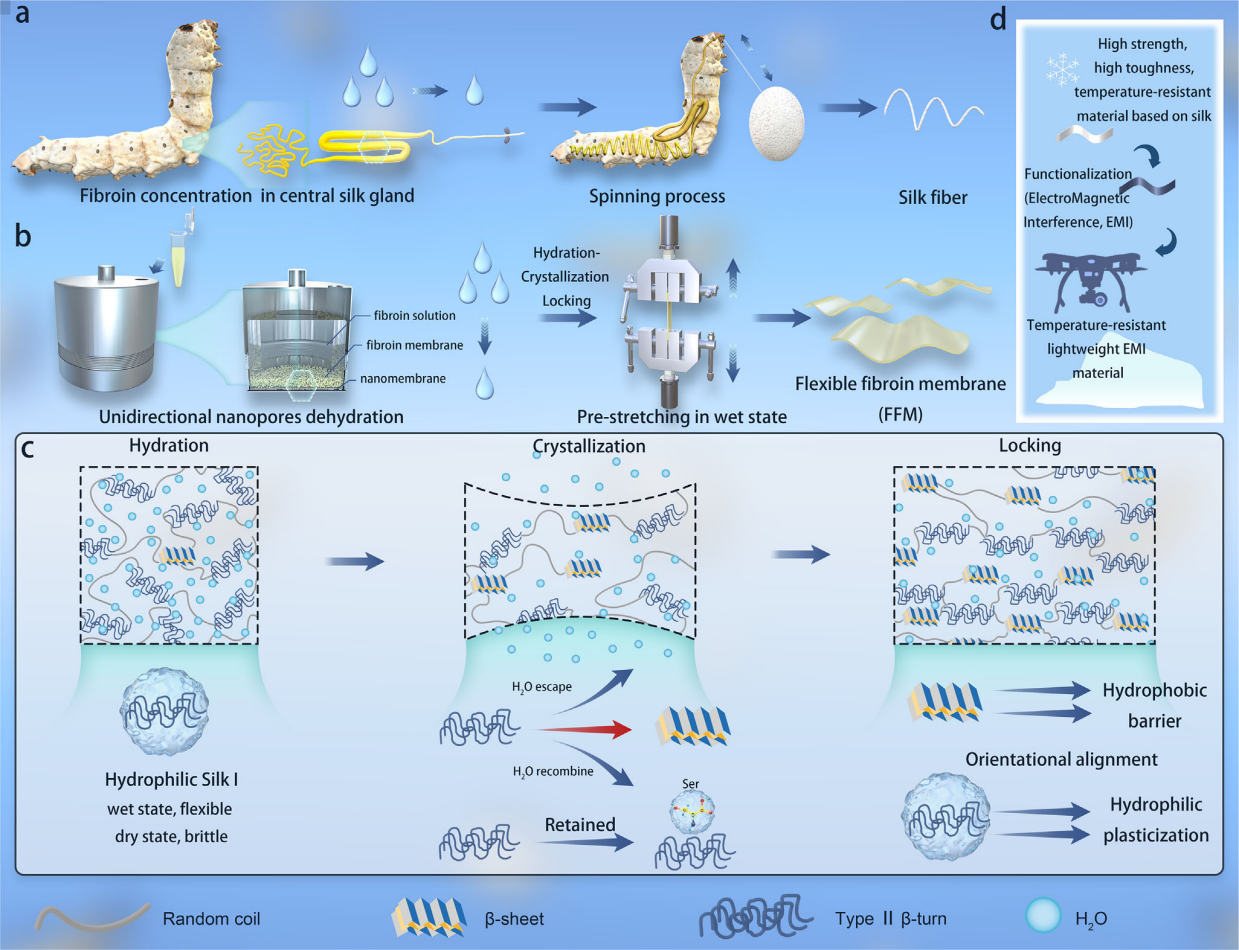
Design and preparation of FFM. (a) In the *B. mori* middle silk gland, silk fibroin undergoes dehydration and concentration, then is stretched through the spinneret to form a silk fiber. (b) Mimicking the *B. mori* spinning mechanism, a regenerated silk fibroin solution is dehydrated and concentrated via unidirectional nanopores dehydration to produce a silk fibroin membrane (UFM). UFM is subsequently highly hydrated and crystallized into an FFM through stretching. (c) Similar to the *B. mori* spinning process, this method initially yields Silk I, which undergoes a structural transition to Silk II, accompanied by dynamic changes in water molecule interactions following the HCL strategy. Specifically, the hydrophilic Silk I structure is highly formed and retained while partially transitioning to the hydrophobic Silk II structure. Silk I in FFM captures water molecules via serine, fixes them to form hydrated structures. Uniformly dispersed Silk II crystalline domains serve as hydrophobic and thermally insulating barriers, preventing water escape and freezing. These hydrated domains, with their ordered orientation, enhance molecular chain plasticization. (d) In extreme temperature conditions, particularly in low‐temperature environments, FFM can function as a high‐performance, temperature‐resistant material based on SF. When integrated with EMI shielding properties, it becomes a lightweight, temperature‐resistant material suitable for use in the outer shells of equipment, such as drones, operating in specialized environments.

Inspired by the *B. mori* spinning process from aqueous fibroin in the silk gland into a fibrous fiber, accompanied by the structural change from Silk I to Silk II (Scheme 1a), we propose an innovative “Hydration‐Crystallization Locking” (HCL) strategy. As illustrated in Scheme 1b, initially, a highly hydrated SF having Silk I structure similar to the concentrated aqueous fibroin in the silk gland was expected by virtue of the directional nanopore dehydration technology [34]. Mimicking the spinning process, pre‐stretching was performed on the hydrated SF to induce Silk II crystal structure and lock the hydrated structure, thereby forming the flexible fibroin membrane (FFM). The HCL strategy depicted in Scheme 1c closely resembles the structural transformation of silkworm spinning. We speculated that serine residues within the hydrophilic segments of the Silk I structure were crucial for capturing water molecules, enabling the formation of a hydrated, plasticized network in silk fibroin membrane created by HCL (Scheme 1c). Simultaneously, the hydrophobic segments of the Silk II structure form crystalline domains that shield the hydrated network. By controlling the interactions between SF molecules and water, we aim to develop SF‐based materials with both high strength and toughness suitable for extreme environments, including extreme‐temperature resistant materials and multifunctional temperature‐resistant platform materials (Scheme 1d).

## 2 Results

### 2.1 Design and Characterization of FFM

**FIGURE 1:**
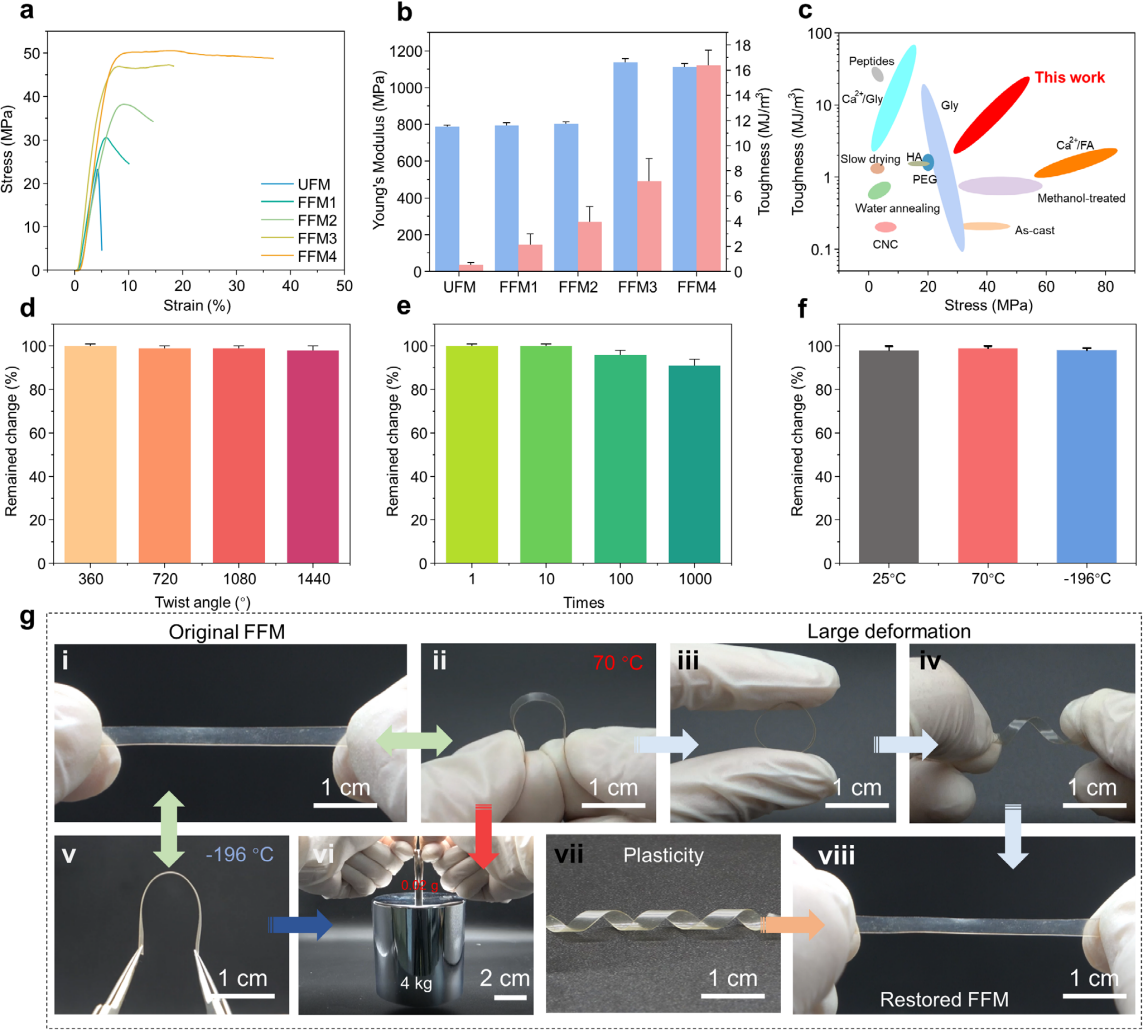
Mechanical characterization of FFM. (a) Stress‐strain curves of FFM, UFM were pre‐stretched with a ratio of 1 to 4, resulting in FFM1 to FFM4; (b) Toughness and modulus of FFM; (c) Various Strength‐toughness diagrams of SF membranes; (d) Ability to deform at large angles of the FFM; (e) Fatigue resistance of the FFM; (f) Fatigue resistance of the FFM in 25°C, 70°C, and −196°C; (g) Macroscopic properties of FFM, including stable flexibility at 70°C (i to ii) and −196°C (i to v), large deformation flexibility (ii to iv, viii), high strength (v to vi, ii‐vi) and plasticity (vii).

Following the HCL strategy, the membranes of FFM1‐4 were successfully fabricated. Compared to the control that was directly obtained by unidirectional nanopore dehydration (UFM), the strength of FFM1‐4 was increased from 31.1 MPa (FFM1) to 50.5 MPa (FFM4), with intermediate values of 38.2 MPa and 46.9 MPa for FFM2‐3 (Figure 1a). Especially, the modulus and toughness of FFM4 reached 1.1 GPa and 16.4 MJ/m^3^ (Figure 1b), respectively. Focusing on the toughness and strength, we found that FFM4 reached the optimal balance compared to the previously reported membranes, such as Glycerin‐SF membrane (Gly‐SFM), Ca^2+^/Formic acid (FA)‐SFM, etc., as illustrated in Figure 1c [35, 36, 37, 38, 39]. Furthermore, after high temperature treatment at 70°C, FFM4 can be bent from a plate to a curved form at a large angle, as illustrated in Figure 1g ii–iv. And the bending angle of FFM4 can be reached from 180°, 360°, 720°, 1080° to 1440° (Figure 1d). In contrast, SF membranes without the HCL strategy were prone to retaining brittle properties (Figure S1). Interestingly, the treatment of dehydration followed by drying enabled FFM4 to have plasticity that has the spiral morphology (Figure 1g vii, viii; Figure S2). Furthermore, such flexibility remained stable, retaining 98% residual deformation after 100 cycles of 180° bending and over 90% after 1000 cycles (Figure 1e). Especially, under temperature from ∼70°C to ∼−196°C, FFM4 exhibited flexibility with 180° bending as illustrated in Figure 1g i, ii, v. And the residual deformation can be reached over 95% (Figure 1f). More importantly, FFM4 maintained high strength under ∼70°C and ∼−196°, lifting an object 200 000 times its weight (∼4 kg) at a thickness of 90 µm and mass of 0.02 g (Figure 1g vi). The above testing results indicate that the HCL strategy is a successful way to obtain SF‐based materials having high strength, toughness, stable flexibility, and plasticity under extreme conditions. The superior mechanical properties and temperature resistance of FFM4 likely arise from molecular structure transformations and dynamic water binding during the HCL strategy; these mechanisms are necessary to be further investigated.

### 2.2 Fibroin Molecular Structure Changes

**FIGURE 2:**
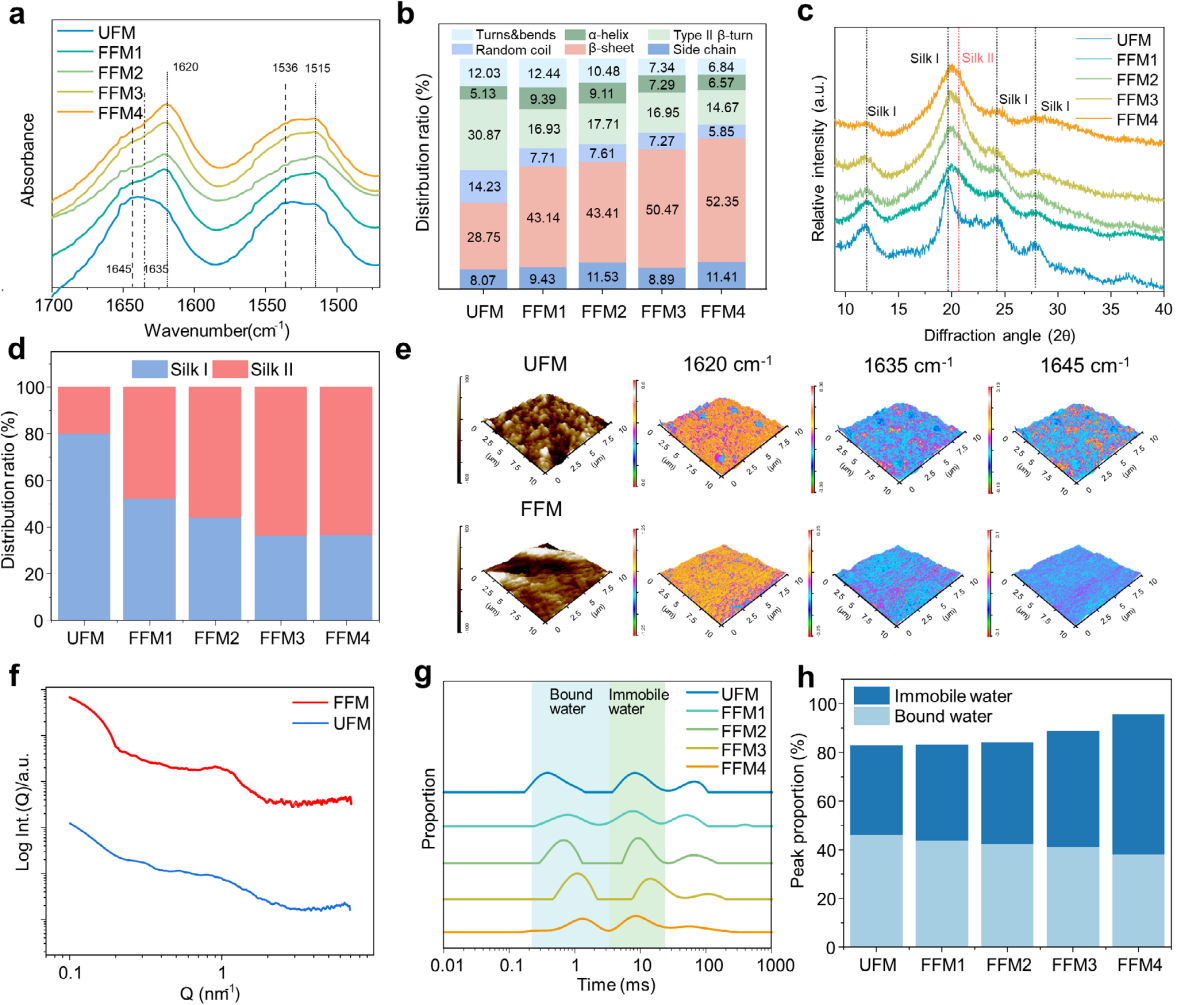
Molecular structural characterization of FFM. FTIR spectrum (a) and secondary structure composition (b) of FFM; XRD pattern (c) and crystalline ratio (d) of FFM;(e) NanoIR images of FFM and UFM; (f) SAXS mapping of FFM and UFM; LF‐NMR analysis (g) and water type changes (h) in FFM.

To investigate how the molecular structure transformations and dynamic water binding impact the mechanical properties and temperature resistance of FFM1‐4, the characterizations, including Fourier‐transform infrared spectroscopy (FTIR), X‐ray diffraction (XRD), Nanoscale infrared spectroscopy (NanoIR), and Low‐field nuclear magnetic resonance (LF‐NMR), were performed. Compared to UFM, FFM1‐4 displayed a shift in the amide I absorption peak from 1645 cm^−1^ (type II β‐turn) to 1620 cm^−1^ (β‐sheet) and increased amide II peak intensity at 1515 cm^−1^ (Figure 2a; Table S1) [40, 41, 42, 43, 44]. The corresponding proportion of type II β‐turn was decreased, accompanied by an increase in β‐sheet from FFM1 2, 3 to FFM4 (Figure 2b; Figure S3). Specifically, for the FFM4, type II β‐turn decreased from 30.8% to 14.6%, while β‐sheet increased from 28.8% to 52.3%. Meanwhile, XRD patterns revealed broadened diffraction peaks of Silk I was shifted from 19.8°to 20.6° (Silk II), companied by other reduced Silk I peaks at 12.2°, 24.7°, 28.2°, 32.3°, 36.8°, and 40.1° [45, 46, 47], and approximately 36.6% of Silk I crystal structure dominated by type II β‐turn was retained (Figure 2c,d). The structural transformation was confirmed by the testing analysis of Raman spectroscopy and ^13^C NMR (Figures S4 and S5) [48, 49, 50, 51]. The above results proved that the HCL strategy induced the structure transformation from type II β‐turn (Silk I) to β‐sheet structure (Silk II) by keeping a reasonable proportion of Silk I. Therefore, the resulting crystalline β‐sheet structure contributed to the mechanical strength of FFM4, and the remained structure, Silk I, was assumed to enable molecular chain plasticization through dynamic water binding to be responsible for the flexibility under extreme conditions.

Thus, the arrangement of hydrophilic structures and their interactions with water molecules of FFM were further investigated. Compared to UFM displaying regional clustering and random dispersion, the intensified peaks of NanoIR in FFM4 at 1645 cm^−1^ indicated that the SF molecular chain arrangement was orderly organized (Figure 2e). Notably, FFM4 exhibited a uniform intensity distribution of the β‐sheet structure at 1620 cm^−1^, probably encapsulating the ordered hydrophilic structures. The observation of small‐angle X‐ray scattering (SAXS) and atomic force microscopy (AFM) revealed that the intermolecular distance of FFM4 was 6.09 nm (Figure 2f; Figure S6), and the surface nanostructures were 150 nm [51, 52], respectively, smaller than those of UFM, proving the tightly packed SF molecular chains of FFM4 to form β‐sheet and encapsulate the hydrophilic structures. Furthermore, the binding forms of water molecules under these molecular distributions were investigated. Thermogravimetric analysis (TGA) showed FFM4 exhibiting a water loss from 11.6% to 8.4% at the thermal decomposition temperature (around ∼ 280°C), which was lower than that of free evaporated UFM (∼6.2%) [53, 54], suggesting the strong binding of high‐affinity water (bound and immobile water) in FFM4 (Figure S7). LF‐NMR further indicated that the bound water signal of FFM1, 2, 3 to FFM4 shifted rightward. Especially, the bound water of FFM4 was decreased from 45.9% to 38.1% and immobile water was increased from 36.9% to 57.5% (Figure 2g,h), reflecting the transition from bound to immobile water as previously reported [55, 56]. The above results confirmed that the HCL strategy can realize the structure transformation of SF together with a dynamic shift from bound water to immobile water by the directional arrangement of the hydrophilic structure. Oriented hydrophilic structures formed hydrated structures, probably by capturing immobile water through specific amino acid residues. The uniform, dense β‐sheet crystalline structure served as a hydrophobic barrier and thermal insulator, encapsulating and locking these hydrated structures, thus preventing water molecule diffusion and ice crystal nucleation, significantly enhancing the mechanical properties and temperature resistance of FFM4.

### 2.3 Interaction of SF Molecular Chains with Water Molecules

**FIGURE 3:**
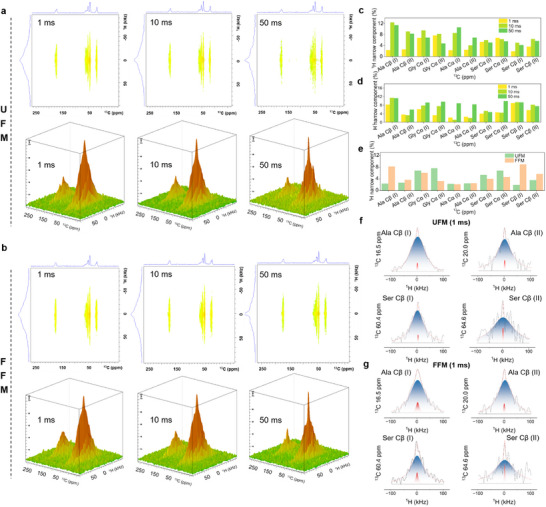
Interaction of SF molecular chains with water molecules. (a and b) ^1^H‐^13^C NMR patterns at 1, 10, 50 ms mixing times; (c) Narrow component changes of UFM at 1, 10, and 50 ms; (d) Narrow component changes of FFM at 1, 10, and 50 ms; (e) Comparison of the narrow components of FFM and UFM at 1 ms; (f and g) ^1^H slices corresponding to ^13^C chemical shifts in the WISE spectrum of FFM and UFM in 1 ms.

To further elucidate the interaction mechanism between specific amino acid residues and water molecules, 2D Wide‐line Separation Nuclear Magnetic Resonance (2D WISE NMR) was employed. As illustrated in Figure 3a,b, differences in ^1^H wide‐line characteristics at various carbon sites, particularly at different mixing times (τm), suggest that specific amino acid residues in UFM and FFM formed unique hydrogen‐bonding interactions with water molecules. The ratio of broad to narrow peak components in ^1^H NMR slices was further analyzed to explore the mobility of water molecules around these amino acid residues (Figure 3c,d; Table S2) [57, 58, 59, 60]. As τm increased from 1 ms, 10 ms to 50 ms, the narrow peak ratios of Gly Cα and Ser Cα in UFM stabilized or decreased, whereas those of Ala Cβ, Ala Cα, and Ser Cβ exhibited an upward trend (Figure 3c). In contrast, the narrow peak intensities of all amino acid residues in FFM increased, suggesting the enhanced molecular mobility was due to the increased water molecule binding (Figure 3d). Notably, at τm = 1 ms, Ala Cβ and Ser Cβ in the Silk I structure of FFM showed narrow peak ratios exceeding 8%, higher than that in UFM by four times (Figure 3e,f). As τm extended to 10 and 50 ms, respectively, the narrow peak ratio of Ala Cβ in FFM approached that of UFM, while the Ser Cβ ratio in FFM rose 1.9 times relative to UFM. Conversely, Ala Cβ and Ser Cβ in Silk II showed no significant increase. These results indicated that FFM enhanced molecular mobility by capturing water molecules through Ser Cβ in Silk I structure to form hydrated structures stabilized by immobile water. The high polarity of serine facilitates water binding and dissociation [61]. These bindings were protected by uniformly dispersed β‐sheet hydrophobic crystalline domains, enhancing the mechanical properties and temperature resistance of FFM.

### 2.4 Potential Applications

**FIGURE 4:**
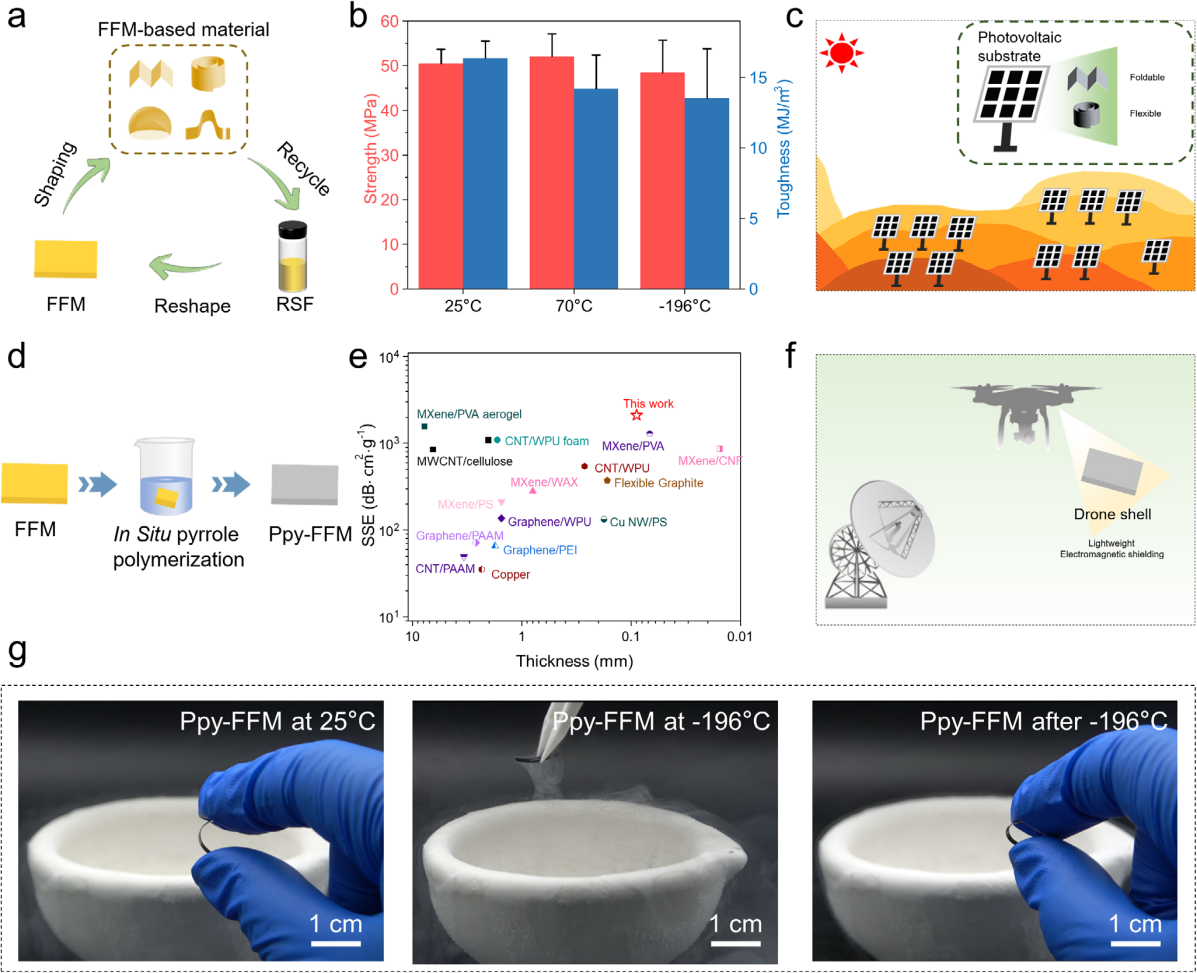
Potential applications of FFM. (a) Plasticity and recyclability of FFM; (b) Mechanical properties of FFM at extreme temperature; (c) The potential of FFM as a flexible photovoltaic substrate; (d) FFM as a pyrrole in situ polymerization substrate; (e) Lightweight electromagnetic shielding performance of Ppy‐FFM; (f) The application potential of Ppy‐FFM as a lightweight electromagnetic shielding material; (g) Flexibility of Ppy‐FFM before and after liquid nitrogen (∼−196°C) treatment.

Based on superior mechanical properties while withstanding extreme temperatures and substantial deformation, the potential applications of FFM in extreme environments were explored. The complex terrain of desert regions, including dunes and Gobi, presents challenges that FFM, as a flexible photovoltaic substrate, can overcome with its programmable deformation capabilities (Figure 4a,c) [62]. FFM maintained a tensile strength of 52.1 MPa and toughness of 14.2 MJ/m^3^ at 70°C (Figure 4b). Its bending properties allowed photovoltaic components to effectively resist sand and wind intrusion, while dynamic deformation aided in the settling of surface sand particles (Figure 4a,c) [63, 64]. Furthermore, following continuous UV irradiation (0.89 W/m^2^, λ = 340 nm) for 168 h, FFM exhibited only a minimal decrease of 1.96% in β‐sheet content and retained 95.7% of its initial tensile strength (Figure S8). Even after exposure to 100% relative humidity for 7 days, the material maintained a tensile strength exceeding 31.9 MPa and retained more than 82.3% of its original toughness (Figure S8). These characteristics enabled better adaptation to rugged landscapes, facilitating the construction of large‐span, high‐clearance photovoltaic power stations in deserts and addressing the installation and application challenges faced by traditional rigid supports. Notably, FFM materials can be recycled under mild conditions (65°C, 3 h) using a ternary solvent (CaCl_2_/H_2_O/EtOH) (Figure 4a; Figure S9), and the recycling solution can be remolded. The recycled FFM exhibited 96.8% retention of its original tensile strength after a single recycling cycle (Figure S10). Even after three recycling cycles, the recycled FFM retained more than 88.1% of its original performance, demonstrating excellent recyclability and strong potential for sustainable applications. This process offers an eco‐friendly solution for the large‐scale development and application of environmentally adaptable materials. Furthermore, in low‐temperature environments at −196°C (Figure S11), FFM demonstrated a tensile strength of 48.5 MPa and toughness of 13.5 MJ/m^3^, as well deformation recovery rate of over 99% after bending. This characteristic suggested potential applications for the housing of polar detection equipment and the spherical shells of polar exploration robots, which required high strength and temperature resistance, as well as adaptive deformation angles ranging from 0° to 180° for navigation across complex terrains.

Additionally, FFM can be used as a platform material for high‐performance temperature‐resistant materials, which are functionally enhanced through simple post‐processing. The polypyrrole‐FFM (Ppy‐FFM) composite was synthesized through *in‐situ* polymerization (Figure 4d). SEM observations (Figure S12) confirmed that Ppy was uniformly distributed within and on the surface of the FFM, forming a continuous conductive network with the conductivity σ = 481.99 S/m (Figure S13). Notably, this composite material achieved a high electromagnetic shielding efficiency (SE) in the X‐band (Figure S14), especially the specific electromagnetic shielding efficiency (SSE) as high as 2083.3dB·cm^2^·g^−1^ (Figure 4e), indicating its potential as a lightweight electromagnetic shielding material. More importantly, Ppy‐FFM retained the temperature resistance characteristics of FFM and allowed large deformation after −196°C treatment (Figure 4g). This performance was expected to be applied to the electromagnetic protection layer of equipment such as drones in special environments (Figure 4f).

## 3 Conclusions

Inspired by the *B. mori* spinning, we developed the “Hydration‐Crystallization Locking” strategy to precisely control the composition and distribution of hydrophilic and hydrophobic domains in SF. This strategy yielded an optimal balance of tensile strength (∼50.5 ± 3.2 MPa) and toughness (∼16.4 ± 1.2 MJ/m^3^), as well as the shape retention over 95% under large deformations at −196°C and 70°C. It was confirmed that the hydrophilic Silk I structure of SF was formed and retained through serine to stabilize immobile water and form hydrated structures, which facilitates molecular chain plasticization. Silk I partially transitioned to the hydrophobic Silk II structure, which was uniformly dispersed to prevent water escape and freezing. HCL‐based FFM exhibited promise for flexible photovoltaic substrates, outer shells for polar exploration equipment, and a platform material in extreme environments. Moreover, FFM was recyclable under mild conditions, providing an eco‐friendly solution for scalable adaptive material development. This work overcame the limitations of temperature‐resistant materials, achieving a balance between mechanical strength, toughness, and temperature tolerance, while providing a method to elucidate the temperature adaptation mechanisms of SF. Crucially, by utilizing renewable SF to replace petroleum‐based polymers while surpassing their performance limits, this work established a sustainable framework for designing next‐generation polymers in extraterrestrial equipment, low‐temperature systems, and multifunctional applications.

## Conflicts of Interest

The authors declare no conflict of interest.

## Supporting Information

Materials and methods; Bending resistance of various silk fibroin membranes; Plasticity of UFM and FFM; Deconvoluted spectra with Gaussian‐Lorentzian fitted curves; Raman spectra of UFM and FFM; ^13^C NMR of UFM and FFM; Atomic force microscope images of UFM and FFM; Thermogravimetric analysis of UFM and FFM; Environmental stability of FFM; Recycling and reuse of FFM; Recycling performance of FFM; Potential application of FFM in polar exploration; SEM image of Ppy‐FFM; Resistance and conductivity of Ppy‐FFM; EMI shielding performance and power coefficients of FFM; Deconvoluted amide I peak positions and secondary structure assignments; 2D WISE NMR chemical shifts of UFM and FFM.

## Supporting information




**Supporting File**: advs73636‐sup‐0001‐SuppMat.docx.

## Data Availability

All data are available in the main text or the supplementary materials.
