# Supplementary material for: Tough and Temperature‐Resistant Material Based on Bombyx mori Silk Fibroin
Source: Adv Sci (Weinh). 2026 Jan 15;13(14):e20165. doi: 10.1002/advs.202520165 (PMC12970169; doi:10.1002/advs.202520165)
Supplement: Supplementary file 1 — Supporting File: advs73636‐sup‐0001‐SuppMat.docx. [file ADVS-13-e20165-s001.docx]

Supporting Information

**Tough and** **temperature-resistant material based on *Bombyx mori* silk fibroin**

Meng Zhang^1^, Quan Wan^1^, Yajun Shuai^1^, Qi Wu^1^, Jing Yu^1^, Mingzheng Fang^1^, Yuqing Zhang^2^, Chuanbin Mao^3^, Mingying Yang^1*^

^1^ Key Laboratory of Silkworm and Bee Resource Utilization and Innovation of Zhejiang Province, Institute of Applied Bioresource Research, College of Animal Sciences, Zhejiang University, Hangzhou 310058, China

^2^ Silk Biotechnology Laboratory, School of Biology and Basic Medical Sciences, Soochow University, Suzhou 215123, China

3 Department of Biomedical Engineering, The Chinese University of Hong Kong, Sha Tin, Hong Kong SAR, China

*Corresponding author. Email: yangm@zju.edu.cn

**1. Experimental section**

**1.1 Materials**

*B. mori* silkworm cocoons were purchased from the Huzhou Academy of Agricultural Science (Zhejiang, China). Na_2_CO_3_, LiBr and glycerin were purchased from Macklin (Shanghai, China).

**1.2 Preparation of regenerated SF solution**

The preparation of regenerated SF solution has been described in detail in previous research ^[1]^. In brief, *B. mori* cocoons were boiled in 0.02 mol⋅L^−1^ Na_2_CO_3_ solution for 30 min to remove sericin. This process was repeated twice and then washed with deionized water. Then the SF fibers were dissolved in 9.3 mol L^−1^ LiBr solution at 60 °C for 3 h. The solution was dialyzed in deionized water for 96 h. Finally, the solution was centrifuged at 8000 rpm at 4 °C for 20 min to remove silk aggregates.

**1.3 Preparation of SF membrane**

UFM was fabricated by the unidirectional nanopores dehydration of silk fibroin solution as reported before by the authors^[2]^. In brief, 3.0 wt.% SF solution was added to the mold, which contains a mold cup (inner diameter of 30 mm), a nanopore film (8-14 kDa, pore diameter of 0.25 nm), a fixed ring, and a rubber stopper. The mold was placed on a horizontal mold stand at 25 °C and 30% humidity. Guided by gravity and the one-way flow of water molecules, SF molecules congregate on the surface of the nanopore membrane, self-assembling into a Silk I-dominated membrane, designated as the Unidirectional-dehydration Fibroin Membrane (UFM). Before pre-stretching, UFM were fully hydrated for at least 24 h, then stretched at 10 mm/min using a testing machine (AGS-J, Shimadzu, Japan) and a 50 N capacity load cell. UFM were stretched between 1−4 times their initial length using the displacement control method with a gauge length of 10 mm, and then statically equilibrated to room temperature for 24 hours, which were labeled FFM1 to FFM4. Ppy-FFM preparation: The FFM was immersed in an aqueous solution containing pyrrole (8% v/v), citric acid (0.02 M), and sodium sulfosalicylate (0.02 M), followed by stirring for 2 h to allow sufficient monomer infiltration. Subsequently, FeCl_3_ solution (1 M) was added to initiate in situ polymerization, which was carried out at −20 °C overnight. After polymerization, the resulting Ppy-FFM was thoroughly washed with deionized water and ethanol to remove unreacted reagents and byproducts.

**1.4 Mechanical properties test**

The mechanical properties of silk fibroin membranes were determined using a universal testing machine (AGS-J, Shimadzu, Japan) at 25 °C and 30% humidity, with a 50 N capacity load cell. Specimens with an initial gauge length of 10 mm were stretched at a rate of 10 mm/min. The bending performance of silk fibroin membranes were tested by bending tools, followed by vacuum drying at 70℃ to remove most of the free water, thereby simulating moisture loss or restriction under extreme conditions. Fix one end of the SF membranes, and the other end bended with the movable rod. With 0° as the initial angle, record the angle change of the bended end.

**1.5 Structure Characterization**

Fourier transform infrared (FTIR)spectroscopy and X-ray diffraction (XRD) patterns analysis refer to the author’s recent report[2]. The OMNIC software was used to perform Fourier self-deconvolution (FSD) on the infrared spectrum covering the amide I area (1595−1700 cm^−1^). The deconvolution used the Lorentz function, the half-bandwidth is 0.964 cm−1, and the noise factor is 1^[3]^. Curve fitting was performed on the FSD spectrum, and the relative area of each component in the amide I area was measured. The Raman spectra of UFM and FFM were obtained using a confocal Raman microscope (LabRAM HR800; Horiba, Jobin-Yvon, France). The excitation wavelength was 785 nm, the slit width was1000 μm, the exposure time was 50 s, and the Raman scattering spectrum range was 200−2000 cm^−1^. To investigate the ordered structure of FFM, a small-angle X-ray scattering (SAXS) system (Anton Paar SAXSess mc2, Austria) with wavelength λ = 0.1542 nm, Cu target, 40 kV, 50 mA was used for scanning. A thermogravimetric instrument (SDT2960; TA Instruments) was used to analyze the thermodynamic properties. The TG curves of FFM are obtained with nitrogen as the protective gas, a test temperature range of 30−800 °C, and a heating rate of 15 °C/min. The nano-infrared test used a multifunctional nanoIR spectrometer (nanoIR2-fs, Anasys Instruments, America) to detect radiation absorption at the nanoscale through the atomic force microscope needle tip, and obtain a component distribution map at a specific wavelength (1620 cm^−1^, 1635 cm^−1^, and 1647 cm^−1^). The low-field nuclear magnetic resonance imager (mesoMR23-060v-I, Suzhou NIUMAG Analytical Instrument Company, China) was used to analyze the water classification type and content changes in the silk fibroin membrane. The ^13^C-^1^H WISE test was carried out using a 500MHz nuclear magnetic resonance spectrometer (AVANCE NEO 500, Bruker, Germany) to analyze the interaction between silk molecules and water molecules. The test conditions are set to: spinning rate 4.5 kHz, recycle delay 3.99s (=3*T1),192 scans, 72 points, spin diffusion 1, 10, 50 ms, chemical shift reference: Adam 38.5ppm.

**2. Supplementary Figures**

**
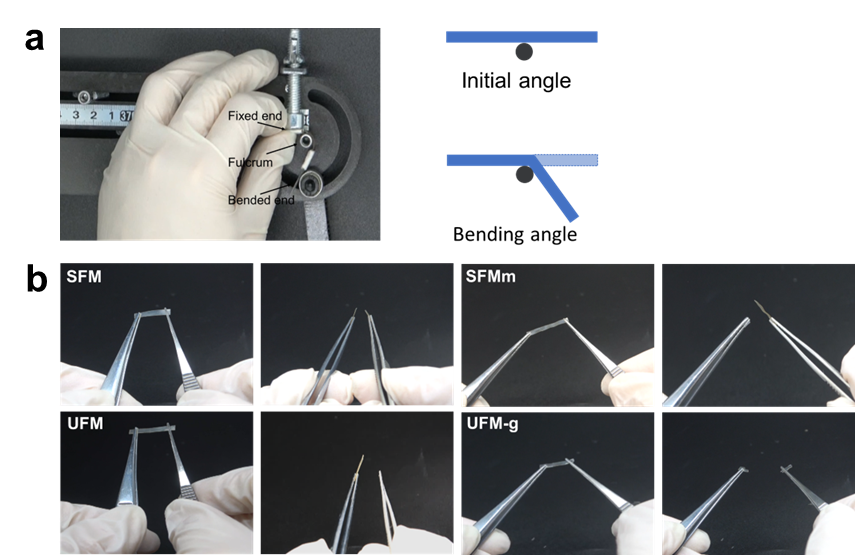
**

**Figure S1.** Bending resistance of various SF membranes. a, bending test tools including fixed end, fulcrum and bended end. Fix one end of the SF membranes, and the other end bended with the movable rod. With 0° as the initial angle, record the angle change of the bended end. b, the brittleness of different SF membranes. SFM: air-dried SF membrane; SFMm: methanol treated SFM; UFM: SF membrane obtained through unidirectional nanopores dehydration; UFM-g: UFM blended with glycerin


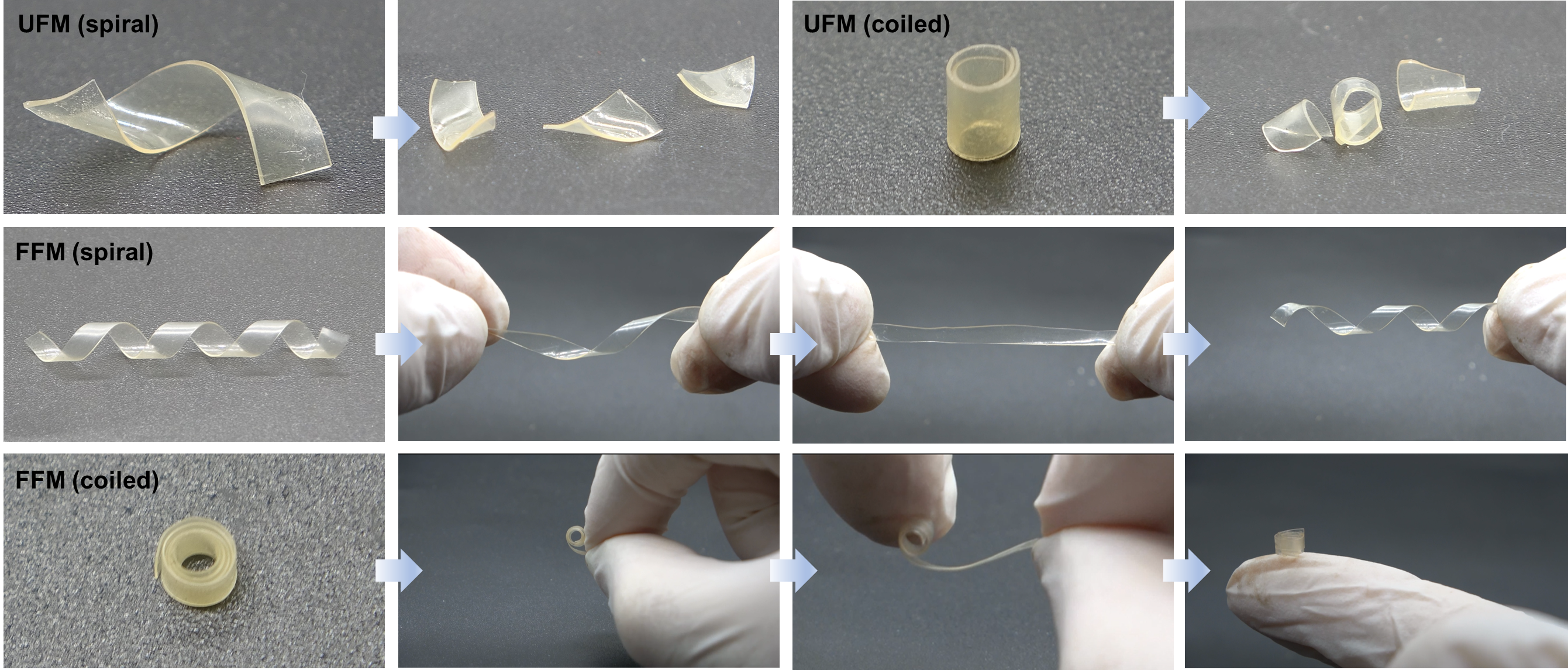


**Figure S2.** Plasticity of UFM and FFM. UFM and FFM were shaped into spirals and curls, respectively. UFM break after being forced, while FFM expand and recover after unloading.


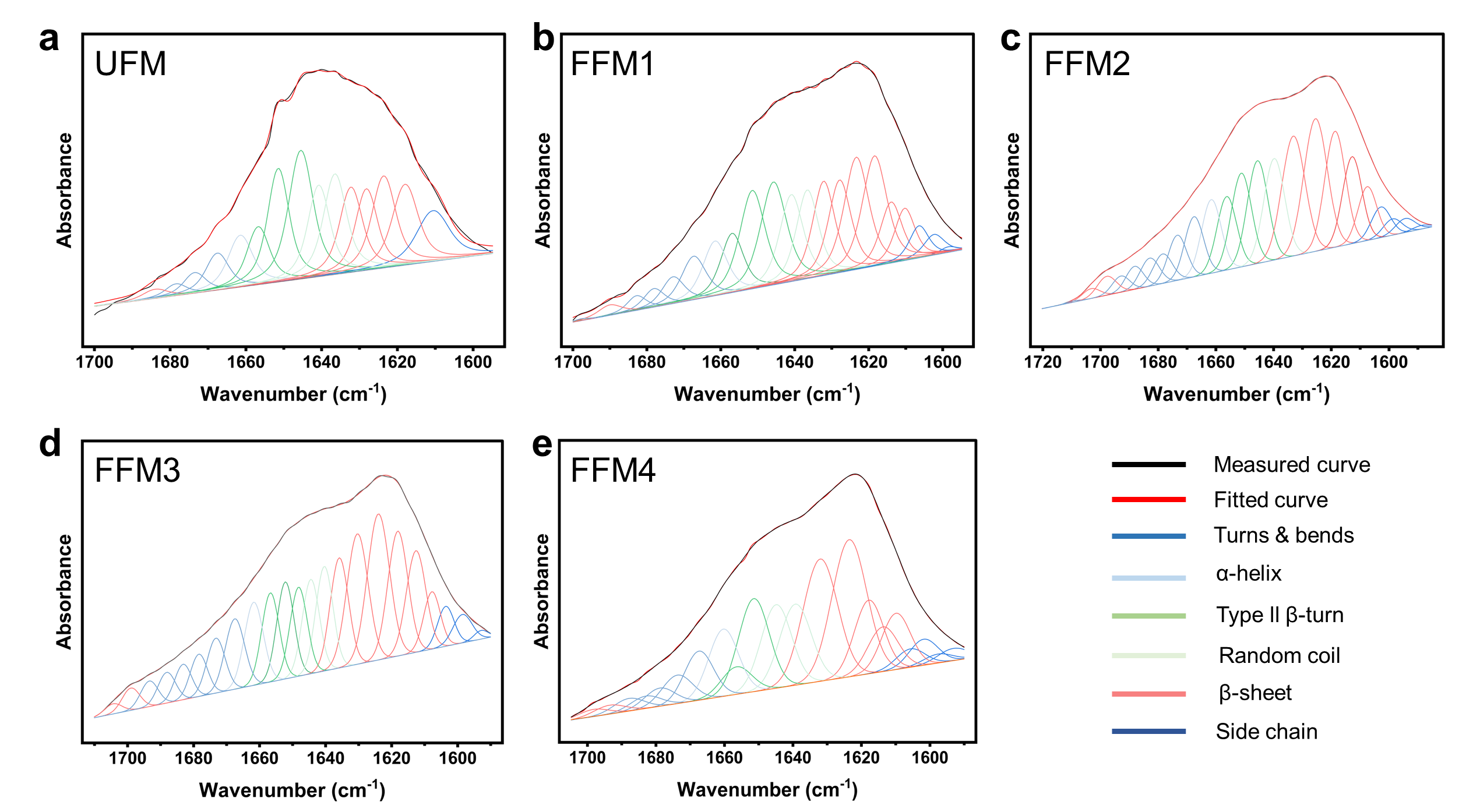


**Figure S3.** Deconvoluted spectra of FFM1-4

**
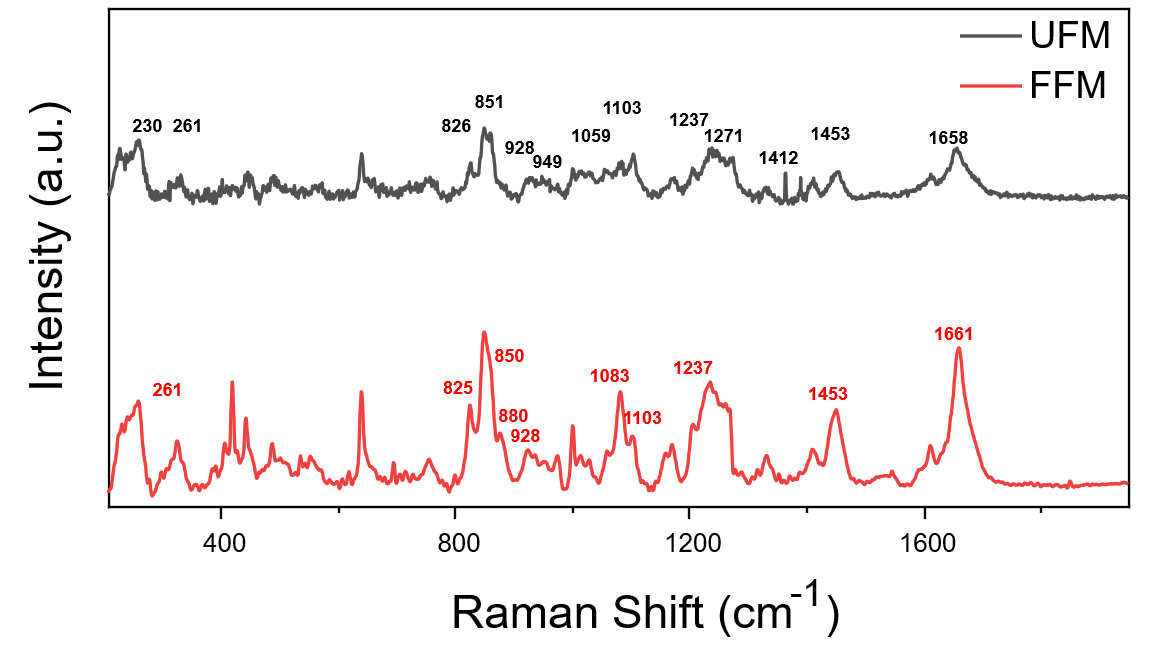
**

**Figure S4.** Raman spectra of UFM and FFM. UFM exhibits Raman marker bands indicative of the Silk I structure at 1658 cm⁻¹ (amide I), 1411 cm⁻¹ (CαH_2_ group vibration), 1273 and 1237 cm⁻¹ (amide III), 1059 cm⁻¹ (νCC backbone, attributed to CC stretching mode), and 850 cm⁻¹ (attributed to tyrosine residues) in the spectral range. In contrast, FFM shows major high-intensity scattering peaks around 880 cm⁻¹, 1083 cm⁻¹, and 1663 cm⁻¹, indicating a higher proportion of β-sheet in the FFM crystal structure.

**
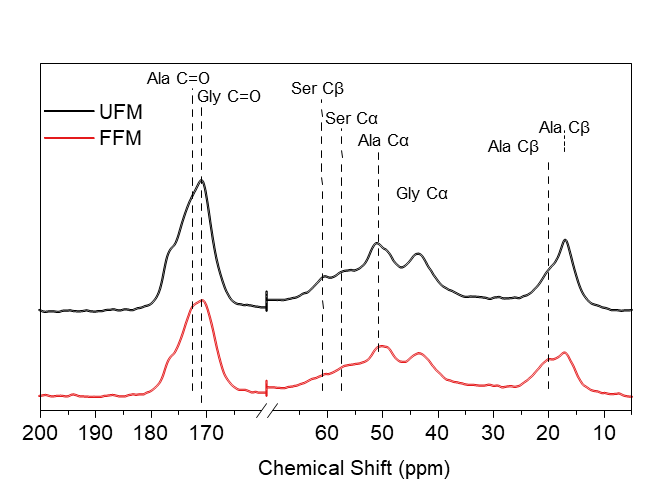
**

**Figure S5.** ^13^C NMR of UFM and FFM. The ¹³C NMR chemical shifts, with primary peaks at 16.5 ppm (Ala Cβ), 52.0 ppm (Ala Cα), and 60.4 ppm (Ser Cβ), indicate that UFM predominantly adopts the Silk I conformation. In contrast, FFM exhibits prominent shoulder peaks at 20.0 ppm (Ala Cβ) and 171.0 ppm (Ala C=O), suggesting the presence of the Silk II structure. Although the 60.4 ppm (Ser Cβ) peak in FFM is less distinct, the 16.5 ppm (Ala Cβ) peak, a critical marker for Silk I, remains well-defined.

**
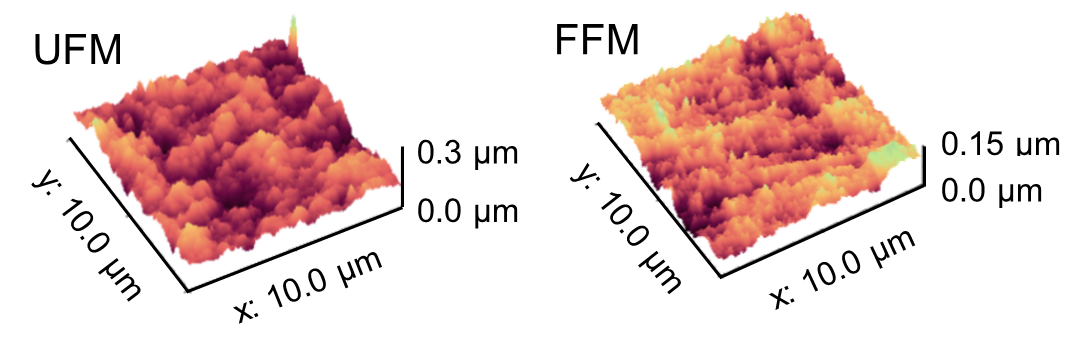
**

**Figure S6.** Atomic force microscope images of UFM and FFM

**
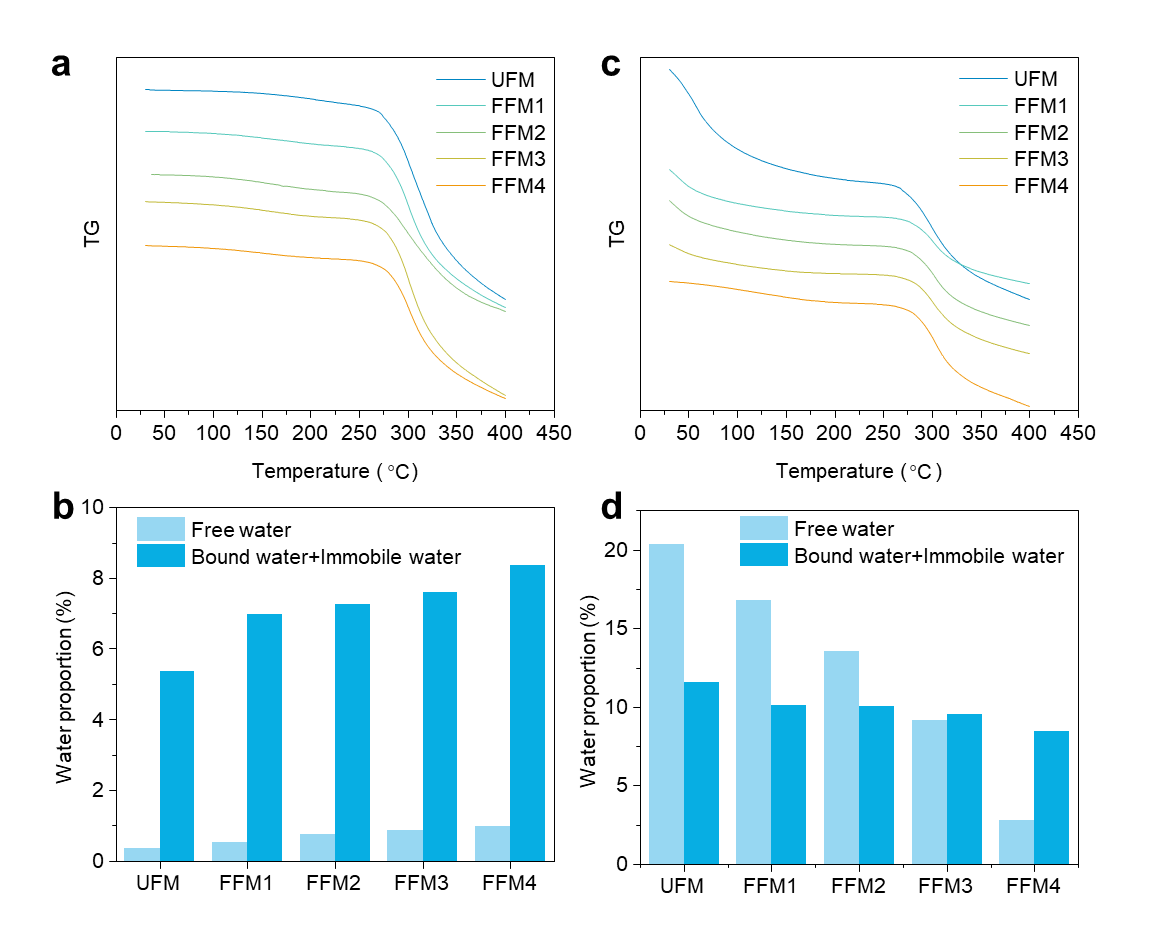
**

**Figure S7.** Thermogravimetric analysis of UFM and FFM. a&b. UFM and FFM in dry state; c&d. UFM and FFM in wet state.


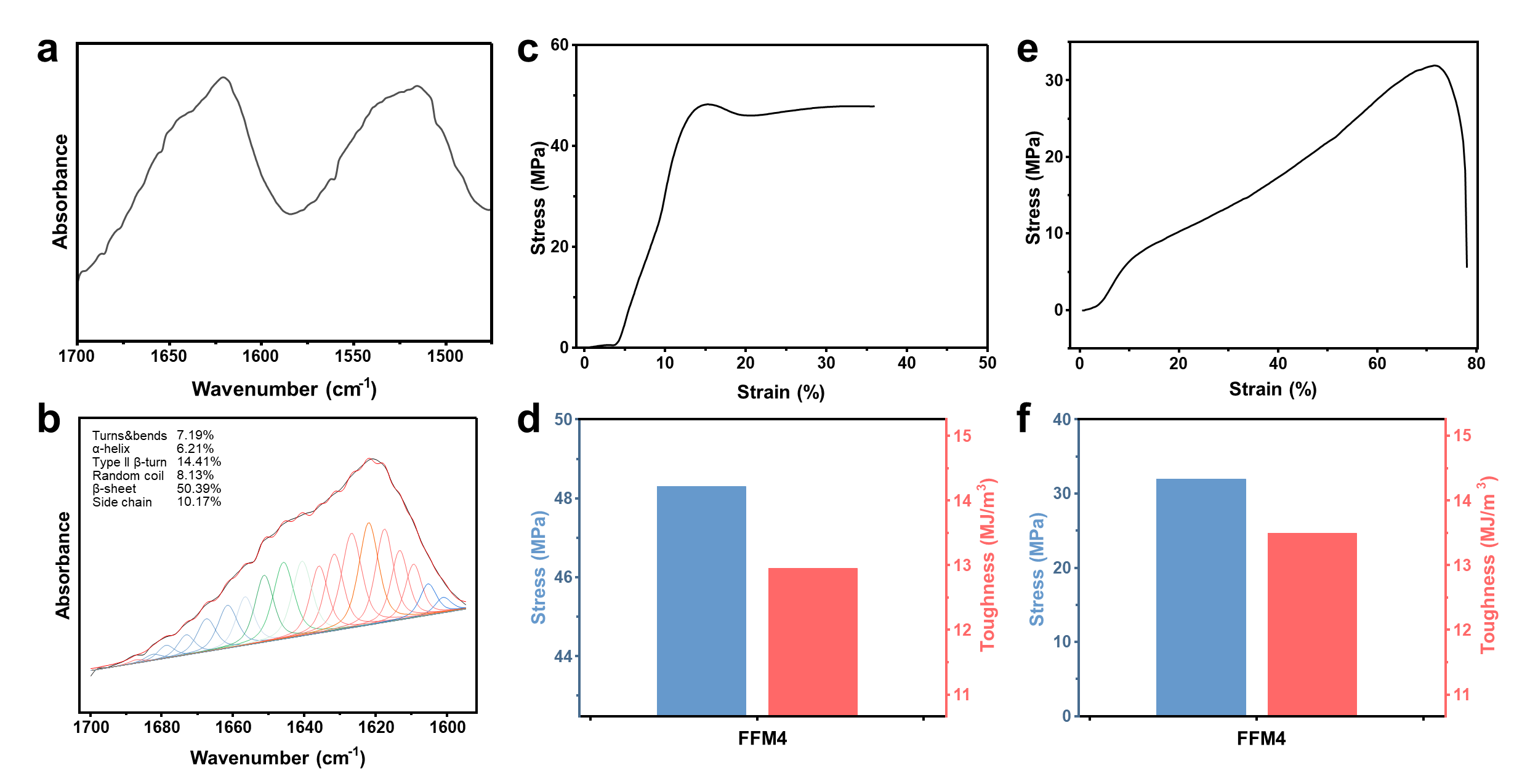


**Figure S8.** Environmental stability of FFM. a. FTIR spectra of UV-exposed FFM; b. Deconvoluted amide I region of UV-exposed FFM; c. Stress-strain curves of UV-exposed FFM; d. Tensile stress and toughness of UV-exposed FFM; e. Stress-strain curves of humidity-exposed FFM; f. Tensile stress and toughness of humidity-exposed FFM


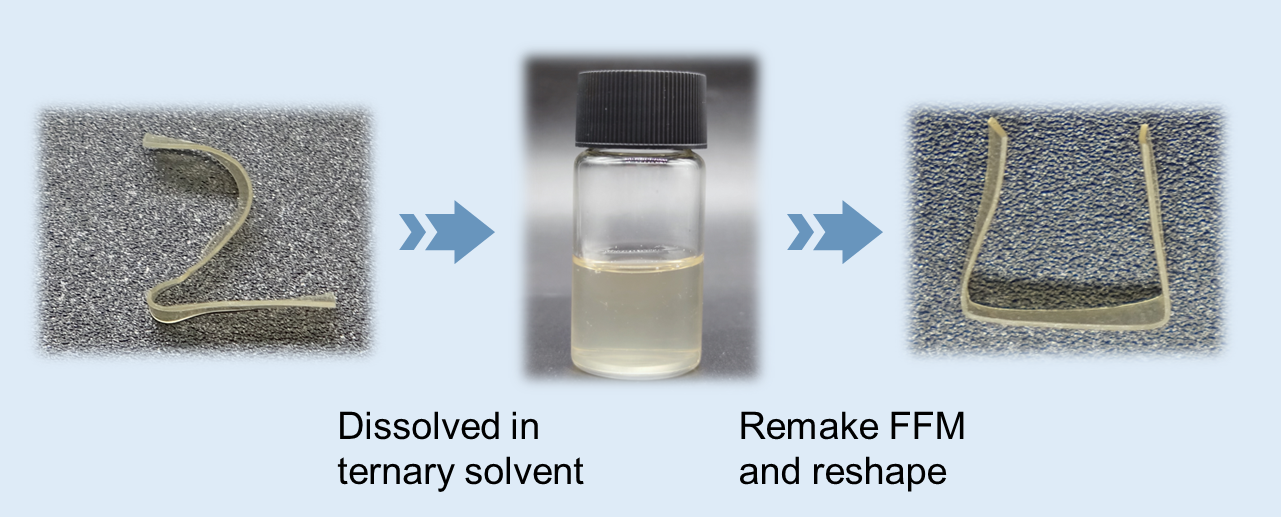


**Figure S9.** Recycling and reuse of FFM


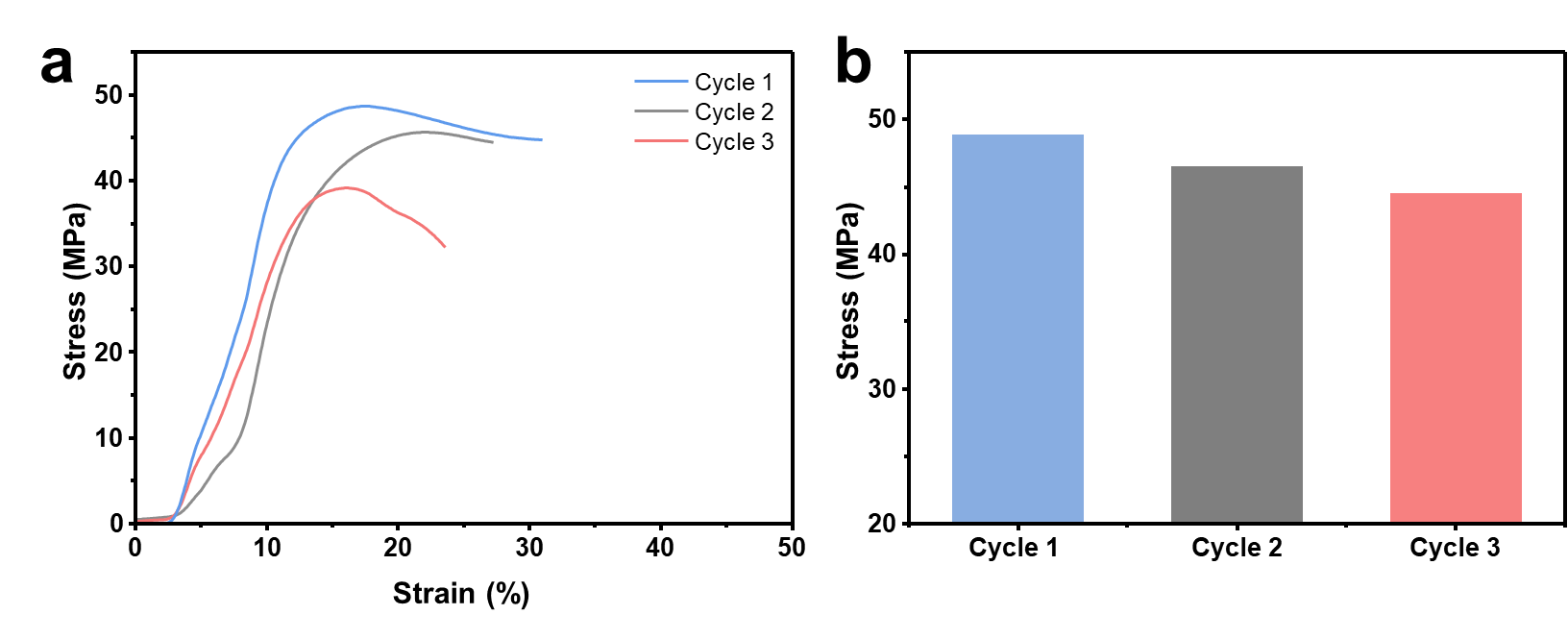


**Figure S10.** Recycling performance of FFM


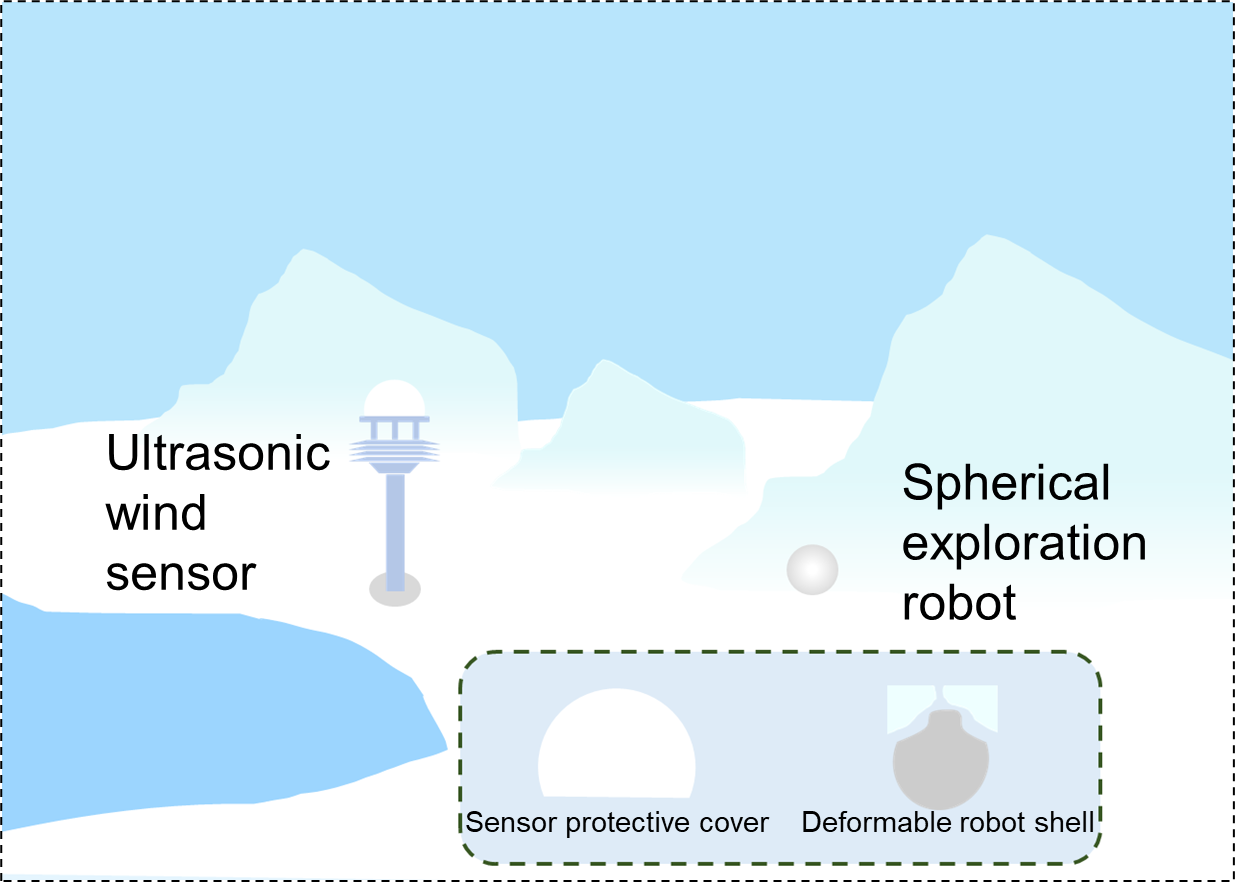


**Figure S11.** Potential application of FFM in polar exploration

**
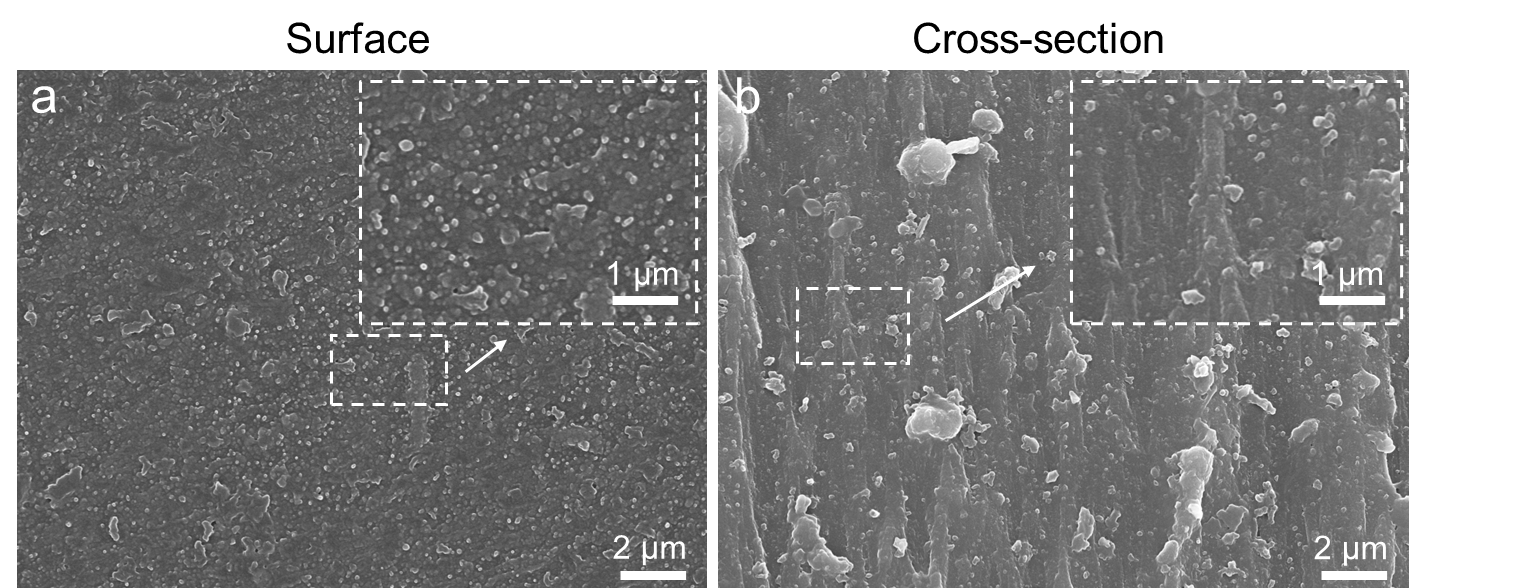
**

**Figure S12.** SEM image of Ppy-FFM. a, the surface of Ppy-FFM; b, the cross-section of Ppy-FFM.





**Figure S13.** Resistance and conductivity of Ppy-FFM

**
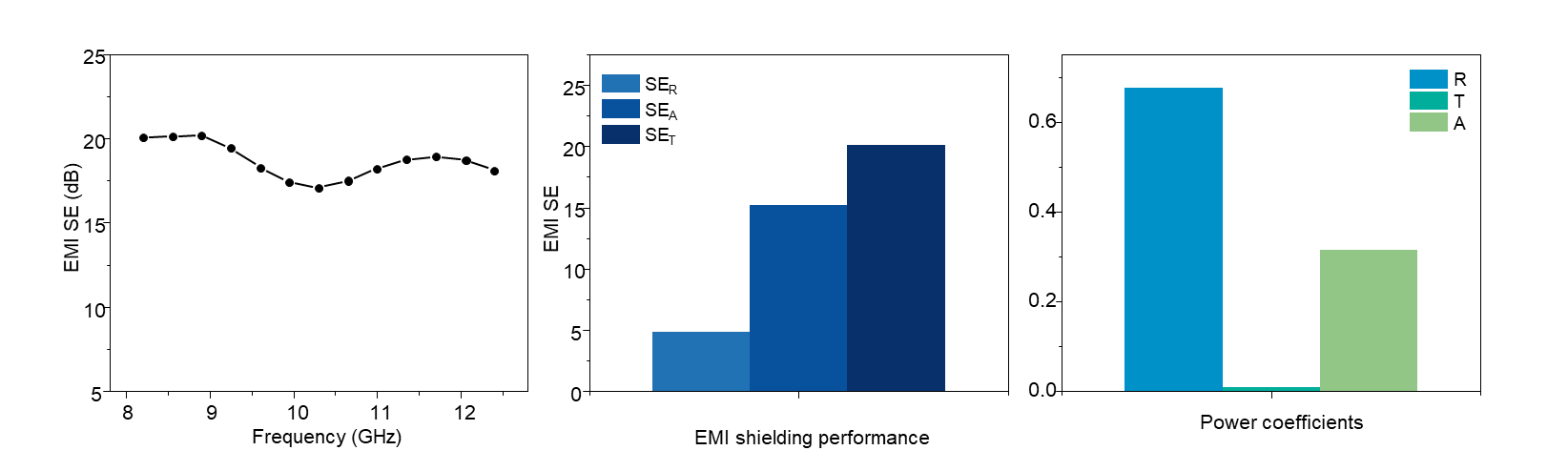
**

**Figure S14.** EMI shielding performance (SE_T_, SE_A_, and SE_R_) and power coefficients (R, T, and A) of Ppy-FFM. SE_T_, SE_A_, and SE_R_ represent total shielding effectiveness, absorption loss, and reflection loss, respectively.

**3. Supplementary tables**

**Table S1.** Deconvoluted amide I peak positions and secondary structure assignments


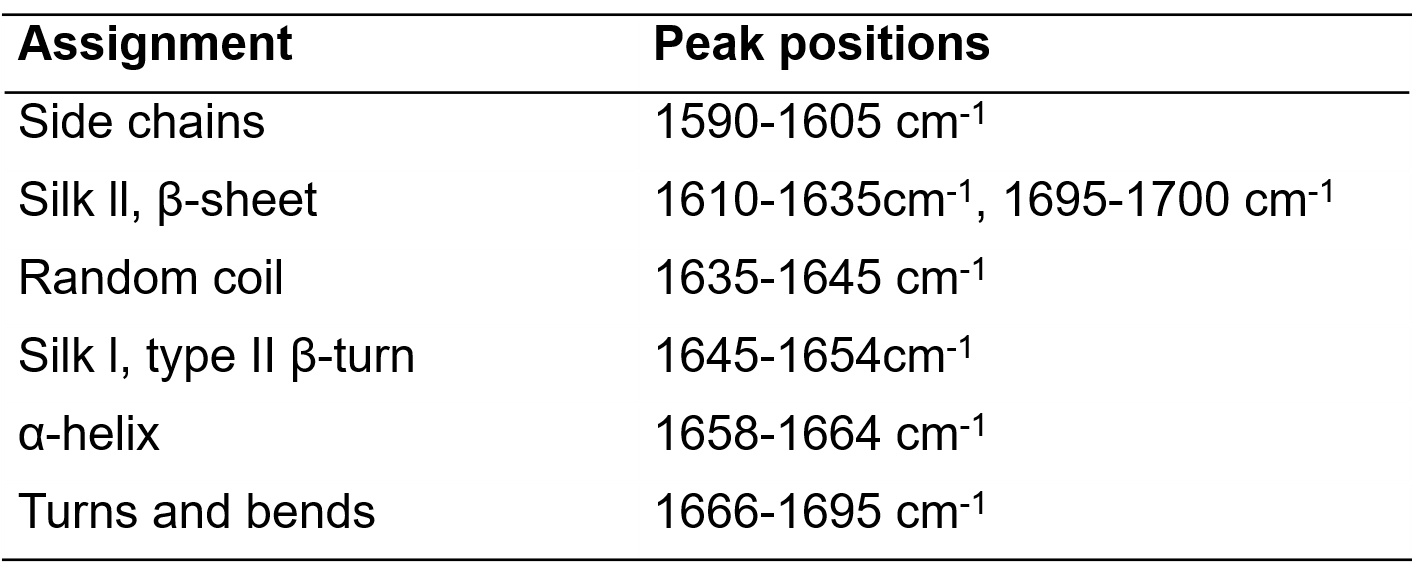


**Table S2.** 2D WISE NMR chemical shifts of UFM and FFM

**
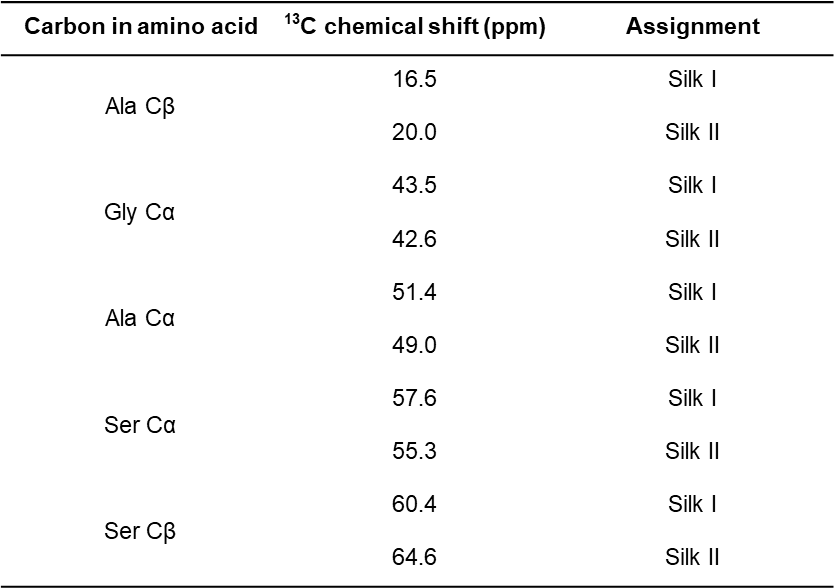
**

**Reference**

[1] Z. Z. Yang, Y. X. You, X. Y. Liu, Q. Wan, Z. P. Xu, Y. J. Shuai, J. Wang, T. B. Guo, J. Q. Hu, J. H. Lv, M. Zhang, M. Y. Yang, C. B. Mao, S. X. Yang, Injectable (Bombyx mori) silk fibroin/MXene conductive hydrogel for electrically stimulating neural stem cells into neurons for treating brain damage, *J. Nanobiotechnol.* **2024**,*22*.

[2] M. Zhang, H. Y. Wang, Y. Q. Zhang, Unidirectional nanopore dehydration induces a highly stretchable and mechanically robust silk fibroin membrane dominated by type II β-turns, *ACS Biomater. Sci. Eng.* **2023**,*9*, 2741.

[3] Q. Lu, X. Hu, X. Q. Wang, J. A. Kluge, S. Z. Lu, P. Cebe, D. L. Kaplan, Water-insoluble silk films with silk I structure, *Acta Biomater.* **2010**,*6*, 1380.
